# Predictors of surgery choices in women with early-stage breast cancer in China: a retrospective study

**DOI:** 10.1186/s12885-023-10510-4

**Published:** 2023-01-06

**Authors:** Sijia Huang, Qingmo Yang, Xujuan Zheng, Ka Ming Chow, Junhua Wu, Jiemin Zhu

**Affiliations:** 1 Department of Nursing, School of Medicine, Xiamen University, Xiamen, P.R. China; 2 Department of Breast Surgery, First Affiliated Hospital, Xiamen University, Xiamen, P.R. China; 3 School of Nursing, Health Science Centre, Shenzhen University, Shenzhen, Guangdong Province P.R. China; 4 The Nethersole School of Nursing, Faculty of Medicine, The Chinese University of Hong Kong, Hong Kong SAR, P.R. China

**Keywords:** Breast cancer, Surgical choices, Breast-conserving surgery, Breast reconstruction, Mastectomy

## Abstract

**Background:**

The breast-conserving surgery and reconstruction rate in China is relatively low when compared with those in Western countries. Moreover, predictors of surgical choices for women with breast cancer in China have not yet been explored. This study aims to explore differences in the surgical choices of women with different demographic and clinical characteristics and the predictors that influence surgical choices of women with early-stage breast cancer.

**Methods:**

This retrospective study included women with early-stage (0-II) breast cancer who underwent surgeries at one of two Xiamen University-affiliated hospitals between 2009 and 2017. Using medical records, eleven variables were collected: the woman's age, year of diagnosis, hospital, marital status, payment method, cancer stage, presence of positive axillary lymph node, histology, neoadjuvant chemotherapy, radiotherapy, and the type(s) of surgery they chose. Binary logistic regression was used to analyse predictors of surgical choice.

**Results:**

A total of 1,787 cases were included in this study. Of the total number of women with breast cancer, 61.3% underwent mastectomy without breast reconstruction, 26.4% underwent mastectomy with breast reconstruction, and the remaining 12.2% chose breast-conserving surgery. Women with different demographic and clinical characteristics underwent different types of surgery. Cancer stage, neoadjuvant chemotherapy, radiotherapy, and the choice of hospital were found to be predictors of breast-conserving surgery. Meanwhile, age, year of diagnosis, payment method, neoadjuvant chemotherapy, and the choice of hospital were found to be predictors of reconstruction after mastectomy in women with early-stage breast cancer.

**Conclusions:**

In China, surgical choices for women with breast cancer have diversified. Healthcare workers should understand the surgical preferences of women of different ages. For early detection of breast cancer, knowledge of breast self-examination and breast cancer screening should be provided. Adequate information about the safety of reconstruction and advocacy for medical insurance coverage of reconstruction should be offer. Breast surgeons need specialised training and standardising protocols towards different types of breast surgery. These actions will help women make better, well-informed decisions about their breast surgeries.

## Background

Breast cancer is the most common cancer and causes the highest number of cancer-related deaths among women globally [1]. In 2020, there were 416,371 new cases and 117,174 deaths caused by breast cancer in China [2]. More than 90% of Chinese women diagnosed with early-stage breast (0-II) cancer are treated with either breast-conserving surgery (BCS), mastectomy with reconstruction, or mastectomy without reconstruction [3], all of which have similar survival rates [4]. There is still a large difference in the surgery patterns for breast cancer between China and Western countries. A nationwide study of 110 hospitals in China reported that 22% of women chose BCS, and 10.7% chose breast reconstruction after undergoing a mastectomy [5]. In contrast, 56.4%–64.5% of women with breast cancer chose BCS in the United States [6, 7], and 67.4% did so in South Korea [8]. Both of these rates are much higher than China's. Moreover, the rate of breast reconstruction after mastectomy in the United Kingdom has reached 24%–75% [9].

Since the 1990s, the National Comprehensive Cancer Network (NCCN) guidelines have recommended BCS with radiotherapy for women with early-stage breast cancer [10]. Women who undergo BCS face fewer surgical risks and shorter recovery times than those who choose other surgical treatments [7]. Women who undergo mastectomy may experience breast asymmetry or body disfigurement that is significant enough to be perceptible, which can result in psychological distress [11]. Breast reconstruction and BCS can both minimise body disfigurement, improve mental health, and improve long-term quality of life [12–14].

For women with early-stage breast cancer, decisions regarding which surgery to have are affected by age, clinical characteristics, the availability of radiotherapy, and financial status. Older women are more concerned about the adverse effects of radiation therapy than cosmetic results and tend to prefer mastectomy [6, 15]. Younger women tend to maintain their breast shape through BCS or breast reconstruction [16]. Women with larger tumours, lymph node metastasis, and comparatively advanced clinical stages are often inclined to undergo mastectomy [6]. As radiotherapy needs to be administered alongside BCS, the availability of radiotherapy influences the choice of surgery. The shortage of radiotherapy resources and trained radiation oncologists or technologists contributes to lower rates of BCS in China [17]. Moreover, previous research has shown that the social economy affects the possibility of breast reconstruction; people with medical insurance are more likely to receive breast reconstruction [18].

To our knowledge, few studies have explored predictors of surgical choices in women with early-stage breast cancer in China. Thus, this study is an important addition to the literature because that information greatly assists in identifying strategies to promote BCS and breast reconstruction.

## Methods

### Aims

This study aims to explore the differences in surgical choices made by women with early-stage breast cancer with different demographic and clinical characteristics and to explore predictors that influence a woman's choice in surgery.

The research questions driving this study are as follows:

- (a) What are the trends in the different types of breast surgeries chosen by women with early-stage breast cancer from 2009 to 2017 in Xiamen City?
- (b) What are trends among the different surgical choices made by these women, as categorised by their different demographic and clinical characteristics?
- (c) What are the predictors for undergoing BCS in women with early-stage breast cancer?
- (d) What are the predictors of breast reconstruction in women with early-stage breast cancer who have already had a mastectomy?

### Design

This is a retrospective study.

### Settings

The research data for this study is collected from two hospitals affiliated with Xiamen University. Hospitals XUFAH and XUAWCH contain 43 and 50 beds in their breast surgery units, respectively. As such, hospitals XUFAH and XUAWCH receive annual averages of 500–600 and 300 women with breast cancer, of whom approximately 150 and 50 are residents of Xiamen city. One hospital has radiotherapy resource. The other one does not provide radiotherapy but always refers patients to other hospitals to receive radiotherapy as their routine care.

### Participants

The inclusion criteria for the study are as follows: (1) a diagnosis of early-stage (0-II) breast cancer in participating hospitals from January 2009 to December 2017; (2) Xiamen city residents; and (3) underwent surgery. The exclusion criteria are as follows: (1) men with breast cancer; (2) a co-occurring malignant tumour or fatal disease; (3) the choice not to undergo surgery; and (4) a medical file missing data on surgery.

### Outcomes and measurement

In this study, we collected two types of data: (1) demographic characteristics: age, year of diagnosis, marital status, and payment method; and (2) clinical characteristics: hospital, stage, histology, presence of positive axillary lymph node, surgery information, neoadjuvant chemotherapy, and radiotherapy. In this study, age is collected as a continuous variable; it is categorised, by years of age, into four groups: < 40, 40–54, 55–69, and ≥ 70. The clinical stage of breast cancer is determined by tumour size, lymph node metastasis, and distant metastasis [19].

### Data-collection procedure

The data in this study were obtained from hospital medical records, which were written by the same breast surgeons who performed the surgeries on the sample of women this study examines. Two trained researchers read the medical records, extracted information, entered that information into a data pool, and quantified the data to form a database.

### Data analyses

The researchers used SPSS version 22.0 for data analysis and employed descriptive statistics to summarise important demographic and clinical data. Analysis of variance and the Pearson chi-square test were used to compare the differences in surgical choices for women with different demographic and clinical characteristics. All women' demographic and clinical variables were entered into a binary logistic regression model, and then the Backward LR method analyses the predictors of surgical choices. The variables that contribute significantly to the outcome are presented in the tables. A receiver operating characteristic (ROC) curve was used to determine the accuracy of the model. This study used a two-tailed *p*-value of *p* < 0.05.

## Results

**Fig. 1:**
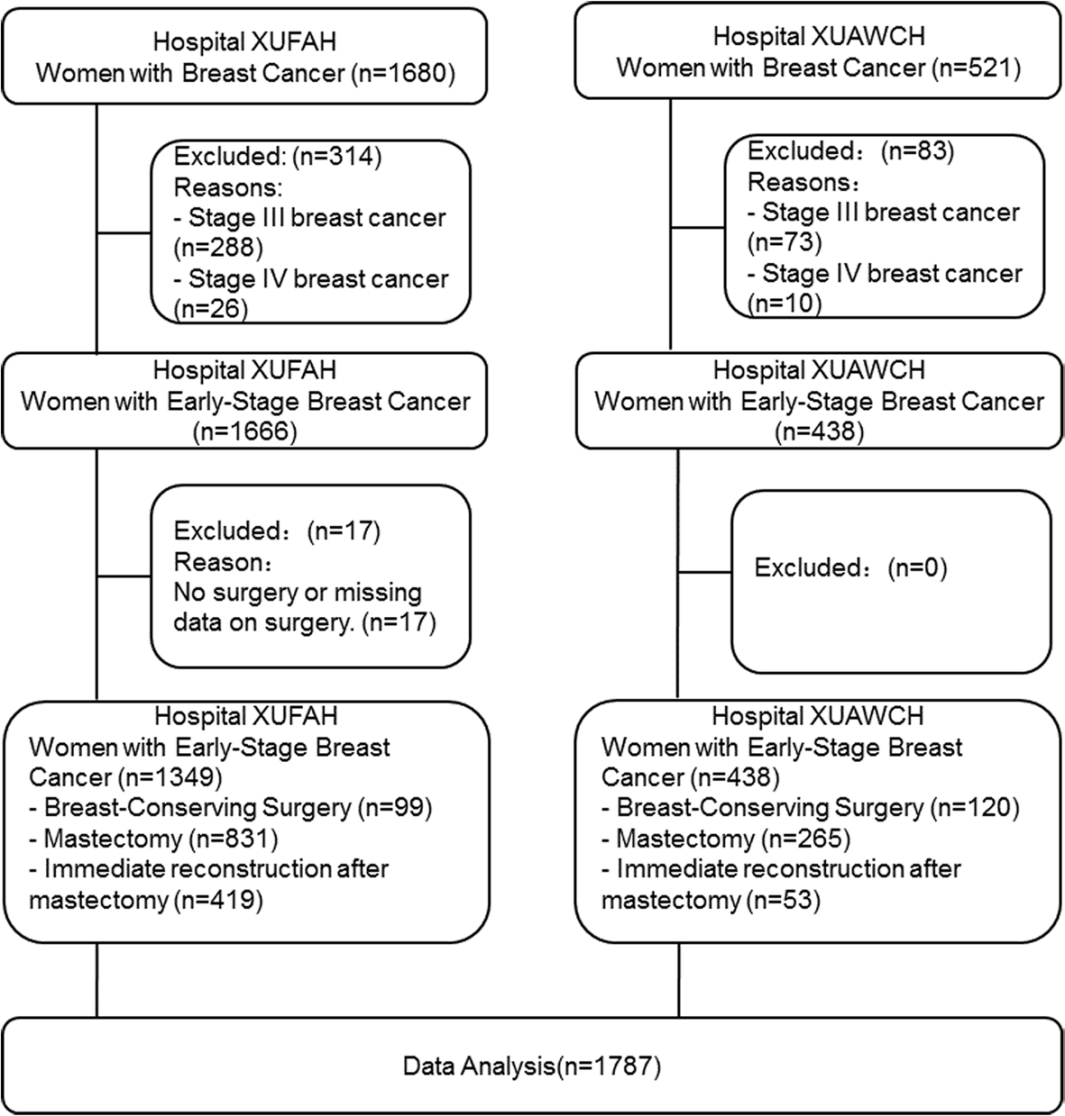
Flowchart of Selection Process. Note: Abbreviation: XUFAH, Xiamen University First Affiliated Hospital; XUAWCH, Xiamen University Affiliated Women and Children's Hospital

The two study hospitals had a total of 2,201 women diagnosed with breast cancer between 2009 and 2017 who were residents of Xiamen city. Of those 2,201 women, 361 were diagnosed with stage III breast cancer, 36 were diagnosed with stage IV breast cancer, and 17 did not receive surgery or had data missing about their surgery. As such, these 414 women were excluded, leaving a total of 1,787 cases to include in the data analysis. The selection process for each hospital is shown in Fig. 1. The majority of participants in our study underwent mastectomy without reconstruction (*n* = 1096, 61.3%), followed by those who opted for a mastectomy with reconstruction (*n* = 472, 26.4%), and then by those who chose BCS (*n* = 219, 12.2%) (Table 1).

**Table 1:** Sociodemographic and clinical data of participants with different surgical procedures (*n* = 1787)

Item	n(%)/Mean (SD)				F/*χ*^2^	P
	**Total**	**Mastectomy without reconstruction (*n* = 1096, 61.3%)**	**Mastectomy with reconstruction (*n* = 472, 26.4%)**	**Breast-conserving surgery (*n* = 219, 12.3%)**		
Age: Mean(SD)^a^	49.37(11.67)	52.88(11.48)	42.50(8.69)	46.64(10.92)	161.989	<0.001
Age groups: n(%)^b^					276.023	<0.001
< 40	348(19.5)	115(6.4)	181(10.1)	51(2.9)		
40–54	896(50.1)	518(29.0)	253(14.2)	125(7.0)		
55–69	441(24.7)	372(20.8)	37(2.1)	32(1.8)		
≥ 70	102(5.7)	90(5.0)	1(0.1)	11(0.6)		
Year of diagnosis: n(%)^b^					170.994	<0.001
2009	111(6.2)	99(5.5)	8(0.4)	4(0.2)		
2010	138(7.7)	114(6.4)	14(0.8)	10(0.6)		
2011	145(8.1)	107(6.0)	25(1.4)	13(0.7)		
2012	172(9.6)	117(6.5)	23(1.3)	32(1.8)		
2013	184(10.3)	110(6.2)	36(2.0)	38(2.1)		
2014	226(12.6)	125(7.0)	60(3.4)	41(2.3)		
2015	251(14.0)	130(7.3)	101(5.7)	20(1.1)		
2016	233(13.0)	127(7.1)	77(4.3)	29(1.6)		
2017	327(18.3)	167(9.3)	128(7.2)	32(1.8)		
Hospital: n(%)^b^					153.616	<0.001
XUFAH^c^	1349(75.5)	831(46.5)	419(23.4)	99(5.5)		
XUAWCH^d^	438(24.5)	265(14.8)	53(3.0)	120(6.7)		
Marital status: n(%) ^b^					14.959	0.001
Married	1723(96.4)	1071(59.9)	443(24.8)	209(11.7)		
Unmarried	64(3.6)	25(1.4)	29(1.6)	10(0.6)		
Payment: n(%) ^b^					11.906	0.003
Medical Insurance	1592(89.1)	954(53.4)	435(24.3)	203(11.4)		
Self-pay	194(10.9)	141(7.9)	37(2.1)	16(0.9)		
Missing Data	1(0.1)					
Cancer stage: n(%) ^b^					54.692	<0.001
0	151(8.4)	71(4.0)	40(2.2)	40(2.2)		
I	680(38.1)	385(21.5)	196(11.0)	99(5.5)		
II	956(53.5)	640(35.8)	236(13.2)	80(4.5)		
Presence of positive axillary lymph node: n(%) ^b^					7.367	0.025
Yes	381(21.3)	255(14.3)	91(5.1)	35(2.0)		
No	1406(78.7)	841(47.1)	381(21.3)	184(10.3)		
Histology: n(%) ^b^					43.762	<0.001
Non-invasive	150(8.4)	70(3.9)	40(2.2)	40(2.2)		
Ductal	1313(73.5)	808(45.2)	367(20.5)	138(7.7)		
Lobular	41(2.3)	26(1.5)	10(0.6)	5(0.3)		
Mucinous	52(2.9)	35(2.0)	7(0.4)	10(0.6)		
Mixed	73(4.1)	45(2.5)	16(0.9)	12(0.7)		
Others	60(3.4)	42(2.4)	13(0.7)	5(0.3)		
Missing Data	98(5.5)					
Neoadjuvant chemotherapy: n(%) ^b^					8.906	0.012
Yes	116(6.5)	86(4.8)	19(1.1)	11(0.6)		
No	1668(93.3)	1007(56.4)	453(25.3)	208(11.6)		
Missing Data	3(0.2)					
Radiotherapy: n(%) ^b^					66.265	<0.001
Yes	175(9.8)	74(4.1)	47(2.6)	54(3.0)		
No	1612(90.2)	1022(57.2)	425(23.8)	165(9.2)		

^a^ Analysis of variance test with F value presented

^b^ Chi-square test with *χ*^2^ value presented

^c^ XUFAH, Xiamen University First Affiliated Hospital

^d^ XUAWCH, Xiamen University Affiliated Women and Children's Hospital

### Trend of different types of surgeries from 2009 to 2017

**Fig. 2:**
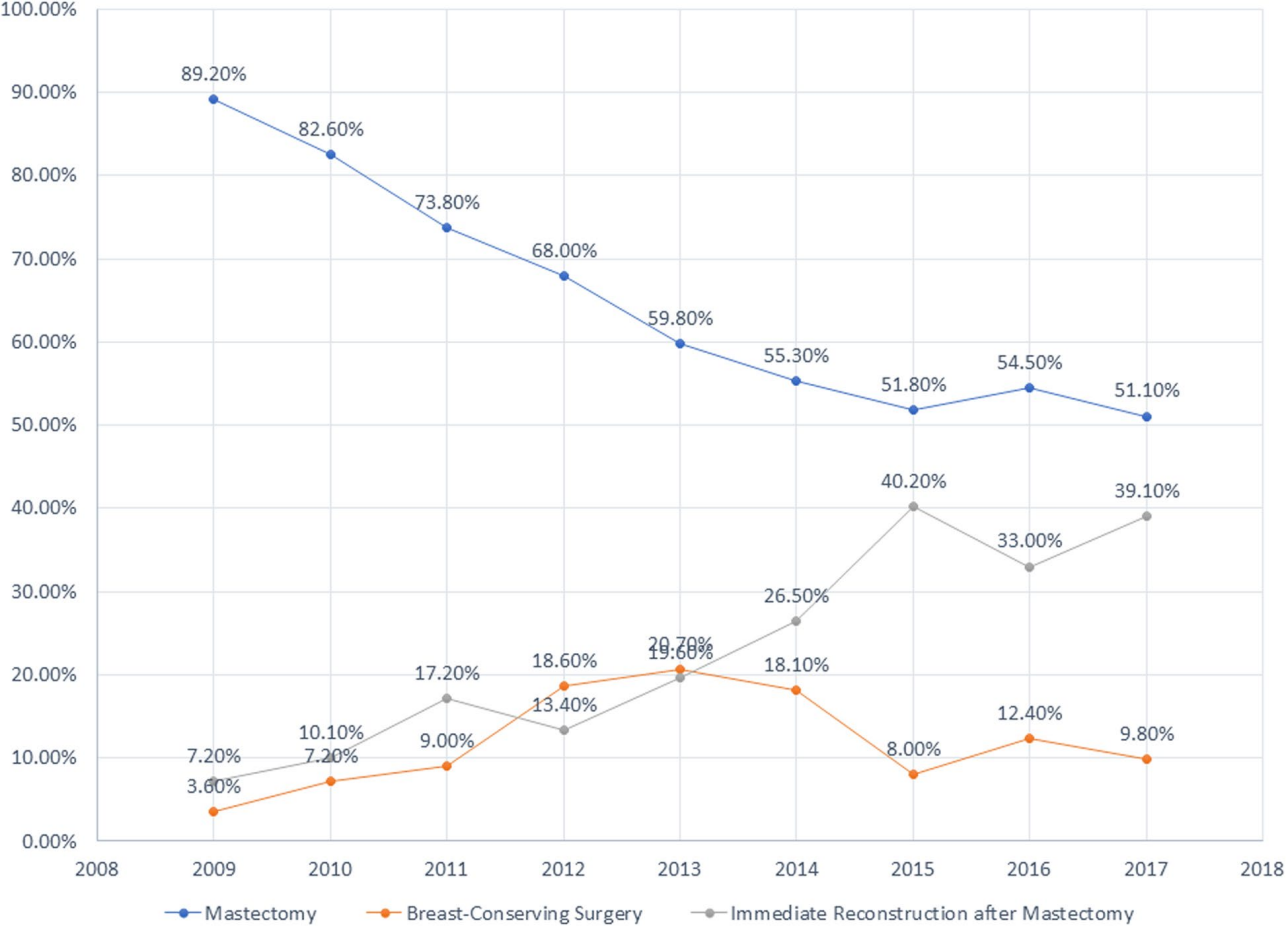
Trend of different types of surgeries for participants with early-stage breast cancer in Xiamen from 2009 to 2017

Between 2009 and 2017, the distribution of different types of surgery changed dramatically. The results are shown in Fig. 2. Since 2009, the proportion of participants undergoing mastectomy without reconstruction has decreased significantly. In 2009, 89.2% of participants with early-stage breast cancer underwent mastectomy without reconstruction. This proportion dropped to 51.8% in 2015 and continued to fluctuate between 51.1% and 54.5% until 2017.

By contrast, the proportion of participants who choose to undergo immediate reconstruction after mastectomy has increased significantly. The highest rate for immediate reconstruction occurred in 2015, at 40.2%, and has consistently remained above 30% since then.

However, between 2009 and 2017, the BCS rate fluctuated while remaining low. In 2009, 3.6% of eligible participants underwent BCS. From 2010 to 2012, this rate increased significantly before reaching an all-time high in 2013, at 20.7%. Thereafter, the BCS rate decreased again and stabilised, remaining between 8.0%–12.4%.

### Sociodemographic and clinic data of participants who underwent different types of surgeries

The sociodemographic and clinical data of participants who underwent different surgeries are summarised in Table 1. The mean age of participants was 49.37 years. The data shows that most of our participants were between the ages of 40–54, followed by the 55–69 age group. Furthermore, many were married (96.4%), had medical insurance (89.1%), and had a diagnosis of stage II breast cancer (53.5%). Most participants (78.7%) did not have axillary lymph node metastasis. Among our participants, invasive ductal carcinoma was the most common histological type (73.5%). Only 6.5% participants received neoadjuvant chemotherapy, and 9.8% received radiotherapy.

Participants with different demographic and clinical characteristics chose different types of surgeries. The participants who underwent mastectomy with breast reconstruction had the lowest average age, followed by those who underwent BCS. The proportion of participants who underwent mastectomy without reconstruction was similar between two hospitals (hospital XUFAH 831:1349, 61.6%; hospital XUACWH 265:438, 60.5%). However, in the remaining cases, participants treated in hospital XUACWH were more likely to choose BCS, whereas participants treated in hospital XUFAH were more likely to undergo reconstruction after mastectomy. Unmarried participants and those with medical insurance were more likely to undergo BCS and mastectomy with breast reconstruction than married participants or those without insurance. The more advanced their breast cancer, the more likely a patient were to choose mastectomy without breast reconstruction. Participants with positive axillary nodes were more likely to choose mastectomy than BCS or reconstruction after mastectomy. Participants receiving neoadjuvant chemotherapy were more likely to choose mastectomy, while participants receiving radiotherapy were more likely to undergo breast conserving surgery and reconstruction after mastectomy.

### Predictors of participants’ choices of different surgical procedures

Table 2 presents the predictors of BCS in participants with early-stage breast cancer. A total of 1475 participants underwent mastectomy with and without immediate reconstruction, while only 210 participants received BCS. Cancer stage, neoadjuvant chemotherapy, radiotherapy, and the choice of hospital were identified as the predictors of BCS, and the accuracy of the model prediction was 81.6%. Furthermore, participants with stage 0 or I breast cancer were more likely to choose to undergo BCS than those with stage II (stage I: [OR = 2.411, 95CI:1.686–3.448, *P* < 0.001]; stage 0: [OR = 4.314, 95%CI:2.635–7.063, *P* =  < 0.001]). Participants received neoadjuvant chemotherapy were less likely to choose BCS (OR = 0.495, 95%CI:0.252–0.972, *P* = 0.041), but those who underwent radiotherapy were more likely to accept BCS (OR = 15.235, 95%CI:9.404–24.681, *P* < 0.001). Additionally, participants treated at hospital XUAWCH were more likely to choose BCS than those treated at hospital XUFAH (XUAWCH [OR = 10.145, 95%CI:6.916–14.883, *P* < 0.001]).

**Table 2:** Predictors of breast-conserving surgery for participants with early-stage breast cancer (*n* = 1685)

Independent variables	β	Standard. error	Wald	Odds Ratio (95%CI)	*P* value
Stage 0 ^a^	1.462	0.251	33.793	4.314 (2.635–7.063)	< 0.001
Stage 1 ^a^	0.880	0.183	23.225	2.411 (1.686–3.448)	< 0.001
Neoadjuvant chemotherapy ^b^	- 0.703	0.344	4.168	0.495 (0.252–0.972)	0.041
Radiotherapy ^b^	2.724	0.246	122.413	15.235 (9.404–24.681)	< 0.001
Hospital XUAWCH ^c^	2.317	0.196	140.449	10.145 (6.916–14.883)	< 0.001

C-statistics = 0.816 (95%CI 0.786–0.846), B = beta coefficient

In binary logistic regression, 0 = mastectomy (including mastectomy with and without immediate reconstruction: *n* = 1,475, 87.5%) and 1 = breast-conserving surgery (*n* = 210, 12.5%)

^a^ Use stage 2 as a control

^b^ Use No as a control

^c^ Use hospital XUFAH as a control

Table 3 presents the predictors of reconstruction after mastectomy in participants with early-stage breast cancer. Age, year of diagnosis, payment method, neoadjuvant chemotherapy, and the choice of hospital were identified as the predictors of reconstruction after mastectomy, and the accuracy of the model's prediction is 83.8%. We found that older participants were less likely to choose reconstruction after mastectomy than younger participants. (OR = 0.890, 95CI:0.876–0.904, *P* < 0.001). The more recently participants were diagnosed with breast cancer, the more likely they underwent immediate reconstruction after mastectomy (OR = 1.367, 95%CI:1.289–1.450, *P* < 0.001). Participants with medical insurance were more likely to choose breast reconstruction than self-paying participants (OR = 1.965, 95%CI:1.247–3.096, *P* < 0.001). Participants received neoadjuvant chemotherapy were less likely to undergo breast reconstruction (OR = 0.536, 95%CI:0.289–0.995, *P* = 0.048). Compare to participants treated at hospital XUFAH, participants treated at hospital XUAWCH were less likely to undergo immediate breast reconstruction after mastectomy (XUFAH: [OR = 0.309, 95%CI: 0.212–0.451, *P* < 0.001]).

**Table 3:** Predictors of mastectomy with reconstruction for participants with early-stage breast cancer (*n* = 1475)

Independent variables	β	Std. error	Wald	Odds Ratio (95% CI)	*P* value
Age ^a^	-0.116	0.008	216.575	0.890 (0.876–0.904)	< 0.001
Year of diagnosis ^b^	0.313	0.030	108.845	1.367 (1.289–1.450)	< 0.001
Payment ^c^	0.676	0.232	8.486	1.965 (1.247–3.096)	0.004
Neoadjuvant chemotherapy ^d^	-0.623	0.315	3.909	0.536 (0.289–0.995)	0.048
Hospital XUAWCH ^e^	-1.174	0.193	37.004	0.309 (0.212–0.451)	< 0.001

C-statistics = 0.838 (95% CI 0.818–0.859), B = beta coefficient

In binary logistic regression, 0 = mastectomy without reconstruction (*n* = 1,022, 69.3%) and 1 = mastectomy with reconstruction (*n* = 453, 30.7%)

^a^ Age was entered as a continuous variable

^b^ Year of diagnosis was entered as a continuous variable from 1 to 9 (eg: the year of 2009 = 1, 2010 = 2……2017 = 9)

^c^ Use self-pay as a control

^d^ Use No as a control

^e^ Use hospital XUFAH as a control

## Discussion

This multicentre study investigates surgical choices of 1,787 women diagnosed with early-stage breast cancer and treated between 2009 and 2017 at one of two hospitals. Our study shows that cancer stage, neoadjuvant chemotherapy, radiotherapy, and the choice of hospital are predictors of a patient's decision to undergo BCS. Moreover, age, year of diagnosis, payment method, whether they undergo neoadjuvant chemotherapy, and hospital are predictors of a patient's decision to have breast reconstruction after a mastectomy.

When compared to an earlier nationwide survey which reported a rate of 5.5% of women undergoing BCS between 1999 and 2008 [3], the 12.3% of women between 2009 and 2017 from our study shows an increase in women receiving BCS. However, a considerable difference still exists between the rates in Xiamen and those of Western countries. The low BCS rate may be partially explained by the conservative attitude that many Chinese breast surgeons have towards BCS, an insufficiency of high-quality training on radiotherapy, and fear of recurrence from both women and surgeons [5].

The overall rate of women from our study who underwent immediate reconstruction post-mastectomy is 26.4%. Moreover, we identified an increase in reconstruction rates between 2009 and 2015, demonstrating an upward trend. In the logistic regression model, year of diagnosis is a predictor of whether women received breast reconstruction after Mastectomy. Our research indicates that an increasing number of women attached importance to their physical appearance or self-image after treatment [18], inducing an increase in their choice for reconstruction after a mastectomy. However, it should be noted that 61.3% of the women who underwent a mastectomy did not pursue breast reconstruction. We find this to be related to the fact that in most Chinese cities, medical insurance does not cover reconstruction treatments [5]. Some women abandoned immediate reconstruction after mastectomy, citing cost-based concerns for implants or mesh [5].

Age is found to be an important predictor of women' surgical decisions. We found that younger women are more likely to choose breast reconstruction after mastectomy, whereas older women tended to stop opting for surgeries after their mastectomy. This is consistent with the results of previous studies [20]. Extant scholarship shows that, for younger women, physical appearance is a major concern and a driving factor related to this trend [21]. When citing reasons for why they chose breast reconstruction, findings offer that younger women do so to improve their self-image, remove clothing limitations, improve relationships, and feel like they overcame cancer [18]. Based on the surgical preferences of women of different ages, this study posits that healthcare workers may apply different approaches when recommending the appropriate surgeries for women from various demographic backgrounds.

In the results, the cancer stage is a clear indicator affecting women' choice of surgery. Women with relatively early-stage breast cancer are more willing to undergo BCS. The cancer stage at diagnosis is also an important factor influencing the women' survival rate: the later the cancer stage, the lower the survival rate [22]. According to Obeidat's research, the cancer stage also affects women's views regarding their cancer and their attitude towards potential risks to their health and survival [23]. This may have a relevant effect, for example, causing women with stage II to be more likely to choose mastectomy than those with stage I breast cancer, as it reduces potential risks to their health, survival, and recurrence of the disease [23]. Chinese women are more conservative [24]. Many wait to visit a doctor until there are palpable breast lumps, and in many of them, the symptoms and tumours are already present when they are diagnosed [24]. Many studies have shown that breast cancer screening aids earlier diagnoses and reduces associated mortality rates [25, 26]. However, in 2010 the overall rate of breast cancer screening in China was very low, with only 21.7% of women reporting that they participated [27]. Thus, this research encourages that breast self-examination knowledge should be offered and breast cancer screening should be promoted among the public. This will assist in detecting symptoms at early stages, thus improving the survivability of breast cancer and, subsequently, the rate of women that opt for BCS.

This study finds that a patient's payment method impacts their tendency to choose breast reconstruction. Women with medical insurance are more likely to receive breast reconstruction than those without insurance, and the difference is significant between the two subcategories. Huang's research suggests that women who undergo breast reconstruction have longer hospital stays, leading to higher out-of-pocket costs for their surgery [16]. Currently, the cost of breast reconstruction in China is approximately 30,000–40,000 yuan, and medical insurance does not cover the cost of prostheses [5]. Greenup's research suggests that, for people with lower incomes, out-of-pocket expenses take precedence over maintaining their appearance [28]. Women with greater out-of-pocket medical expenses may go into debt if they opt for breast reconstruction surgery [29]. Additionally, there is a connection between a breast cancer diagnosis and a reduction in income caused by a reduction in working hours, which increases economic pressure on the patient and their family [30]. These factors inhibit and may prohibit self-paying women from being able to consider breast reconstruction procedures. Thus, due to the many patient benefits associated with reconstruction, we posit that medical insurance should cover the cost of reconstruction, making this option available to women who qualify for and wish to pursue this option in China.

This study also finds that the choice of surgery varies considerably between the two hospitals, and the hospitals become their own predictors of surgical choices in women. In our study, even though one hospital does not provide the radiotherapy, the patients who underwent BCS in this hospital are always been referred to other hospitals nearby to receive radiotherapy as their routine care. So women with breast cancer treated in both hospitals can access to radiotherapy resources. The different attitudes towards various surgeries that breast surgeons in different hospitals have may influence women' decisions regarding their surgery. Some Chinese breast surgeons are more conservative and doubt the therapeutic effects of BCS. Subsequently, breast surgeons with this mindset enforce stricter indications for BCS in their hospitals [5, 20]. Some surgeons do not routinely provide information on BCS to women with breast cancer, leading to lower numbers of BCS in their hospitals. Breast surgeons need to undergo more training to improve their confidence in the efficacy and benefits of BCS; ideally, this will make them more willing to recommend and perform BCS to and on women with breast cancer. Furthermore, if breast reconstruction takes place immediately after a mastectomy for breast cancer, it is mostly performed by the breast surgeon instead of a plastic surgeon, and breast surgeons in different hospitals receive different training in reconstructive surgery [20]. Some breast surgeons may not proactively mention breast reconstruction in women with breast cancer [20]. The effect that a patient's hospital had on their choices in surgery suggests an urgent need to provide more training for breast surgeons regarding the long-term importance of an informed patient choice, as well as ongoing skill training sessions. Similarly, standardising protocols towards different types of surgery in women with breast cancer across hospitals in China could benefit informed patient choice and thus increase BCS and reconstruction rates nationwide.

In our study, only 6.5% women with early stage breast cancer received neoadjuvant chemotherapy. Women who receive neoadjuvant chemotherapy are less likely to receive BCS, which is at odds with the published literatures [31, 32]. Since neoadjuvant chemotherapy can shrink the tumour size and let women who do not meet the requirements of BCS to reach the standard [33, 34], previous studies in western countries reported that the use of neoadjuvant chemotherapy could increase the rate of BCS [31, 32]. Women’ cost-based concerns may explain the difference. In China, low reimbursements still lead to rising out-of-pocket expenses for women with breast cancer, and medical expenditure of cancer treatment can rapidly impoverish families [35]. Neoadjuvant chemotherapy may prolong women’ hospital stay, delay surgery, and increase medical expenses [36, 37]. As a consequence, women who have concern on the financial burden may not consider BCS and reconstruction which require additional costs on radiotherapy or implants respectively, leading to a high rate of mastectomy. In addition, some studies have found that women receiving BCS after neoadjuvant chemotherapy have higher risk of local recurrence [38], which also cause some women to choose mastectomy.

This study finds that only 9.8% women with breast cancer receive radiotherapy, and women who accept radiotherapy are more likely to choose BCS. Radiotherapy is a routine adjuvant treatment of BCS, which can reduce the local recurrence rate after breast conserving surgery [39]. Thus, patient’s intention to receive radiotherapy and the availability of radiotherapy resources may influence the choice of surgery. Chen et al., (2019) believes that radiotherapy is more time-consuming and expensive in comparison with chemotherapy. Women with low income, especially those without medical insurance, may not be able to afford radiotherapy [15]. More radiotherapy resources in China and more comprehensive medical insurance may improve women's ability to accept radiotherapy, which may help to improve the breast conserving rate in China and reduce the risk of local recurrence after BCS.

This study has certain limitations. We only recruited samples from two hospitals in Xiamen, which limits the generalisability of this study. As we collected data from medical records, it is possible that we have missed a few important variables that influence surgical choices, such as education level, personal values, and patient involvement in the decision. Furthermore, we did not assess the role and demographic data of the surgeons involved in these choices, which may influence women's decisions regarding which type of surgery to undergo for their breast cancer [40]. Future studies are warranted to collect more comprehensive data from different regions of China to explore additional predictors of women's choice of surgery, thereby developing strategies to help Chinese women make better-informed decisions regarding which surgery, or surgeries, they want to undergo to treat their breast cancer.

## Conclusion

In China, surgical choices for women with breast cancer have diversified in recent year. The age, year of diagnosis, cancer stage, payment method, choice of hospital, choice of radiotherapy or neoadjuvant chemotherapy all strongly influence the choice of surgery. Healthcare workers should understand the surgical preferences of women of different ages, provide knowledge regarding breast self-examination and breast cancer screening for early detection, offer adequate information about the safety of reconstruction, advocate for medical insurance coverage to cover reconstruction treatment, and receive additional training and standardisation of protocols for different types of breast surgeries available at different hospitals, thus helping women to make better, more informed decisions regarding breast surgeries.

## Data Availability

The datasets generated and/or analysed during the current study are available in the Mendeley repository, found here: https://data.mendeley.com/datasets/bpbn56v3x7/2.

## Abbreviation

BCS: Breast-conserving surgery
